# Supplementary material for: Longitudinal Comparison of Neutralizing Antibody Responses to COVID-19 mRNA Vaccines after Second and Third Doses
Source: Vaccines (Basel). 2022 Sep 3;10(9):1459. doi: 10.3390/vaccines10091459 (PMC9504054; doi:10.3390/vaccines10091459)
Supplement: Supplementary file 1 [file vaccines-10-01459-s001.zip › vaccines-1877290-supplementary.pdf]

Supplementary Appendix

The supplementary appendix has been provided by authors to give readers additional information about the work. Supplement to: Roeder A.J., Koehler M.A., Lake D.F., *et al.* Longitudinal Comparison of Neutralizing Antibody Responses to COVID-19 mRNA Vaccines after Second and Third Doses.

Supplementary Figure S1 is provided to help orient readers with respect to conversion between units described in the main text, such as NAb LFA test line density, percent neutralization, and NAb titer.

NAb LFA Test Image

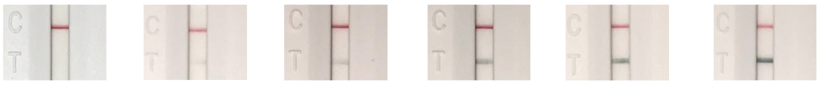

**Test Line Density Units (thousands)**	10-99	100-199	200-369	370-599	600-799	800-1000
**Neutralization (%)**	99-90	89-80	79-61	60-36	35-15	≤15
**NAb Titer Ranges**	<1:1280 ≥1:640	<1:640 ≥1:320	<1:320 ≥1:160	<1:160 ≥1:80	<1:80 ≥1:40	≤1:40

Supplementary Figure S1. Neutralizing antibody later flow assay (NAb LFA) measured in density units corresponds to percent neutralization and NAb titer. Validation of the rapid NAb test using gold standard focus reduction neutralization test ($FRNT_{50}$) with authentic SARS-CoV-2 Wuhan isolate and correlation/regression analyses are previously described [*17*]. Percent neutralization was calculated as 1-Test line density/Limit of Detection (942,481) *100%. Titers are based on the last dilution of a serum sample that inhibited 50% of infectious foci in a Focus Reduction Neutralization test [*17, 18*].

Supplementary Figure S2 demonstrates annexation of sample size with respect to dose and vaccine manufacturer. This flowchart is provided for clarity of sample sizes discussed throughout the manuscript. Figures 3B and 3C in the main text reference "mixed" and "non-mixed" 3rd vaccine doses.

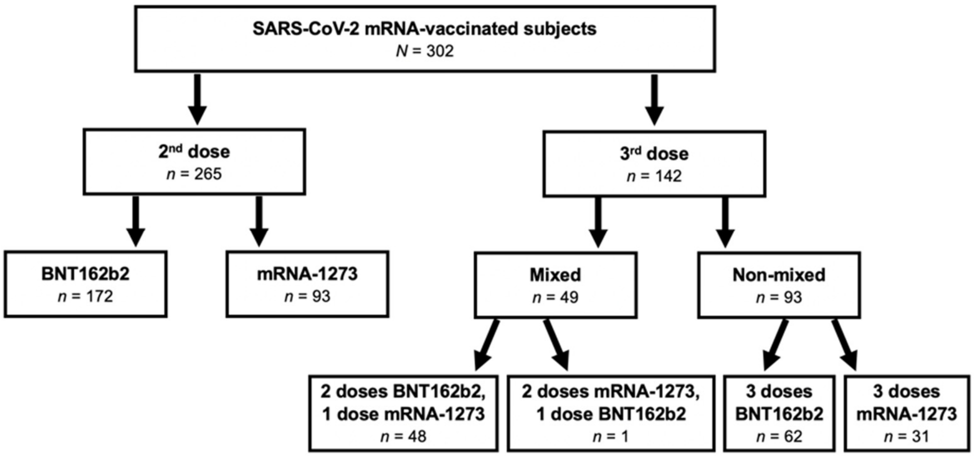

Supplementary Figure S2. Flowchart of mRNA-vaccinated subjects with respect to dose and vaccine manufacturer. Sample sizes for the second dose group are divided into BNT162b2 and mRNA-1273 recipients. The third dose group is first divided into mixed and non-mixed vaccine recipients, and subsequently by number of doses per vaccine manufacturer.

Supplementary Figure S3 shows longitudinal analysis of NAb titers with respect to sex, age, and sex/age concurrently using unpaired 2nd and 3rd dose data sets.

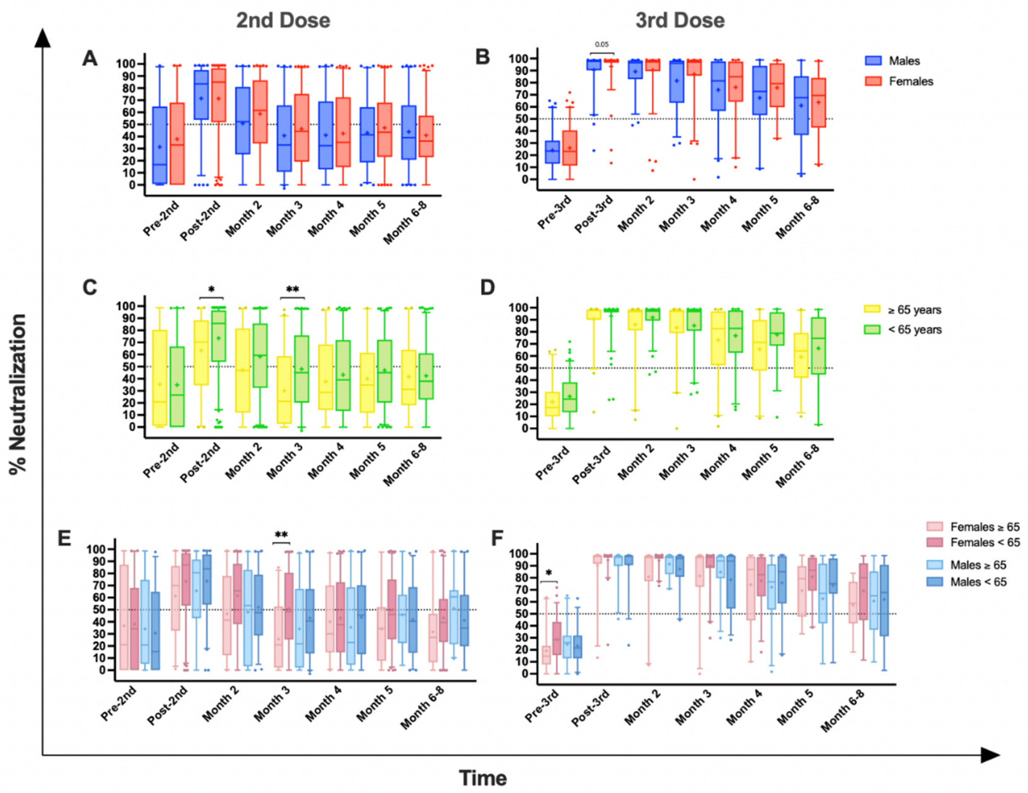

Supplementary Figure S3. Comparison of Second and Third Dose NAb Durability by Sex and Age. Graphs are grouped by column and row. Graphs in the left column represent 2nd dose data, grouped by sex (top row), age (≥ or < 65 years old, middle row), and combined sex and age analyses (bottom row). Graphs in the right column demonstrate 3rd dose data, grouped by row as detailed previously. **A)** Evaluation of sex-based differences after 2nd dose, and **B)** after 3rd dose. **C)** Evaluation of age-based differences after 2nd dose, and **D)** after 3rd dose. **E)** Evaluation of sex and age combined differences after 2nd dose, and **F)** after 3rd dose. Data are shown as grouped box and whisker plots with error bars representing 5th-95th percentile of each population. Outliers outside of the 5th-95th percentile are shown as circular symbols above or below error bars. Data corresponding to graphs in top and middle rows were analyzed using non-parametric Mann-Whitney test to evaluate mean rank between groups with a two-tailed P-value (p<0.05) and 95% confidence interval (CI). Bottom row graphs were generated from data analyzed using a non-parametric Kruskal-Wallis test to evaluate mean rank between groups using multiple comparisons (two-tailed p<0. 05 and 95% CI). Axes are labeled as time (X) and percent neutralization (Y). Pre- and post-vaccine dose timepoints are defined in the (*see* Figure 3 legend).

Supplementary Figure S4 demonstrates a post hoc power analysis of paired longitudinal 2nd and 3rd dose vaccination data, provided in reference to Figure 6 in the main text.

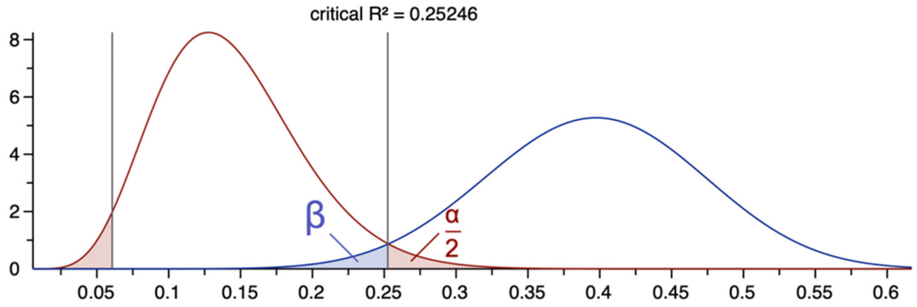

Supplementary Figure S4. Post hoc power analysis of paired longitudinal data set to compute achieved power given α (0.05), sample size (N = 105) and effect size (R^2 = 0.3). Statistic calculated for two-tailed, random-model linear multiple regression (exact test) with 14 predictors: power (1 – β) = 0.974.

Supplementary Figure S5 demonstrates NAb titers prior to breakthrough infection in a population infected prior to the emergence of the SARS-CoV-2 Omicron variant (Delta variant or earlier), compared to a population infected during the Omicron wave.

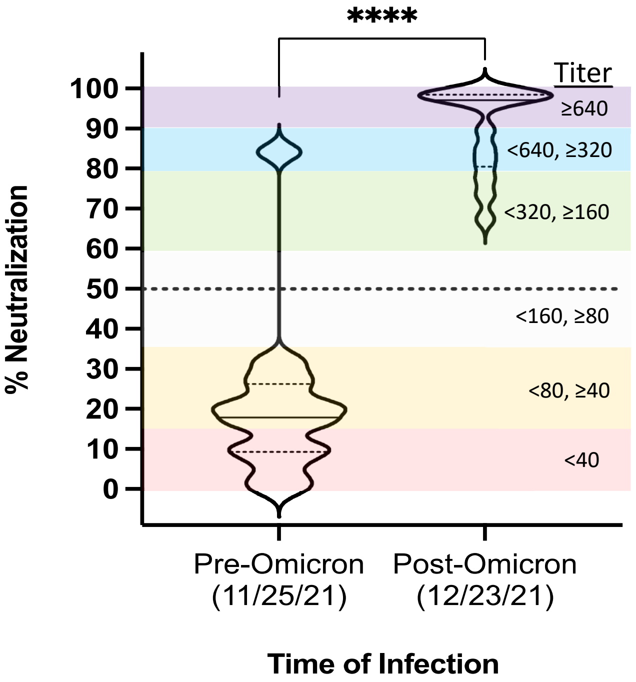

Supplementary Figure S5. NAb titers prior to breakthrough infection in groups infected before and after the onset of Omicron community spread. NAb data prior to infection are shown as percent neutralization violin plots where solid black lines represent median, and dotted black lines indicate upper and lower quartiles. A single outlier in the pre-December 2021 group had 84% neutralization prior to infection, however, was on the 2nd week of a 40 mg/day prednisone taper at the time of infection. Demographic information pertaining to individuals in both groups are detailed in Supplementary Table S1.

Supplementary Table S1 A and B are provided in reference to Figure 7 in the main text, to give readers additional information regarding the time between vaccination, NAb testing, and confirmed breakthrough infection.

A

Age	Sex	1st/2nd Dose mRNA Vaccine	3rd Dose mRNA Vaccine	Date of 3rd Dose	Pre-Infection NAb Test	Pre- Infection % Neut. (NAb Titer)	Date of PCR+	NAb Test to PCR+ (days)
31-35	M	mRNA-1273	mRNA-1273	Nov-2021	Dec-2021	98.9 (>1:640)	Jan-2022	26
61-65	F	BNT162b2	BNT162b2	Oct-2021	Dec-2021	98.8 (>1:640)	Dec-2021	19
56-60	F	BNT162b2	BNT162b2	Oct-2021	Jan-2022	98.8 (>1:640)	Jan-2022	17
46-50	F	BNT162b2	mRNA-1273	Sep-2021	Dec-2021	98.5 (>1:640)	Jan-2022	34
51-55	F	BNT162b2	mRNA-1273	Oct-2021	Dec-2021	98.5 (>1:640)	Jan-2022	27
51-55	F	BNT162b2	BNT162b2	Oct-2021	Dec-2021	98.4 (>1:640)	Jan-2022	20
36-40	F	BNT162b2	mRNA-1273	Nov-2021	Nov-2021	97.4 (>1:640)	Jan-2022	60
46-50	M	BNT162b2	mRNA-1273	Aug-2021	Nov-2021	96.9 (>1:640)	Jan-2022	34
46-50	M	BNT162b2	BNT162b2	Oct-2021	Dec-2021	89.7 (>1:640)	Jan-2022	27
41-45	F	BNT162b2	BNT162b2	Oct-2021	Dec-2021	85.0 (>1:320)	Jan-2022	25
66-70	M	mRNA-1273	mRNA-1273	Oct-2021	Dec-2021	81.6 (>1:320)	Jan-2022	30
61-65	M	BNT162b2	BNT162b2	Aug-2021	Dec-2021	77.6 (>1:160)	Dec-2021	18
41-45	F	BNT162b2	BNT162b2	Oct-2021	Dec-2021	73.4 (>1:160)	Jan-2022	24
36-40	F	mRNA-1273	mRNA-1273	Sep-2021	Jan-2022	67.4 (>1:160)	Jan-2022	6

B

Age	Sex	1st/2nd Dose mRNA Vaccine	Date of 2nd Dose	Pre-Infection NAb Test	Pre-Infection % Neut. (NAb Titer)	Date of PCR+	NAb Test to PCR+ (days)
46-50	M	BNT162b2	Mar-2021	Sep-2021	84.2 (>1:320)	Sep-2021	8
31-35	M	BNT162b2	Jan-2021	Apr-2021	32.3 (<1:80)	Oct-2021	187
26-30	F	BNT162b2	Jan-2021	Jul-2021	28.5 (<1:80)	Aug-2021	18
61-65	M	BNT162b2	Jan-2021	Aug-2021	25.6 (<1:80)	Sep-2021	20
76-80	F	BNT162b2	Jan-2021	May-2021	21.3 (<1:80)	Jul-2021	61
31-35	F	mRNA-1273	Mar-2021	Sep-2021	20.8 (<1:80)	Nov-2021	40
51-55	M	BNT162b2	Feb-2021	Sep-2021	19.4 (<1:80)	Sep-2021	8
51-55	F	mRNA-1273	Feb-2021	Aug-2021	16.6 (<1:80)	Sep-2021	21
76-80	M	BNT162b2	Feb-2021	May-2021	16.3 (<1:80)	Jul-2021	53
51-55	F	mRNA-1273	Feb-2021	Sep-2021	10.8 (<1:40)	Nov-2021	69
56-60	M	BNT162b2	Feb-2021	Oct-2021	10.1 (<1:40)	Oct-2021	3
81-85	F	BNT162b2	Feb-2021	Sep-2021	7.3 (<1:40)	Oct-2021	23
46-50	F	BNT162b2	Feb-2021	Oct-2021	2.9 (<1:40)	Nov-2021	43
41-45	F	BNT162b2	Mar-2021	Aug-2021	0.0 (<1:40)	Sep-2021	17

Supplementary Table S1. Breakthrough infection population demographics. **(A)** Pre-Omicron and **(B)** Post-Omicron population demographics, vaccination status, and NAb result preceding infection. Age, sex, 1st/2nd vaccine dose and date, 3rd dose vaccine and date (if applicable), date of last NAb test prior to infection, pre-infection NAb result, date of positive PCR for SARS-CoV-2 detection, and days between NAb and PCR dates are detailed in these tables. Pre-infection NAb test result is shown as % neutralization, calculated using the limit of detection our rapid NAb lateral flow as described previously Average age of the pre- and post-

December 2021 populations were 54 and 49 (median 55 and 49.5), respectively. Populations in (A) and (B) were 57% and 64% female, respectively.
